# Retraction Note to: Aging-related tumor associated fibroblasts changes could worsen the prognosis of GBM patients

**DOI:** 10.1186/s12935-021-01905-z

**Published:** 2021-04-09

**Authors:** Hongwang Song, Xiaojun Fu, Chenxing Wu, Shouwei Li

**Affiliations:** 1 Department of Emergency Medicine, Shengjing Hospital of China, Medical University, Shenyang, China; 2 Department of Neurosurgery, Sanbo Brain Hospital, Capital Medical University, Xiangshanyikesong 50#, HaiDian District, Beijing, 100093 China

## Retraction Note to : Cancer Cell Int (2020) 20:489 https://doi.org/10.1186/s12935-020-01571-7

The Editors-in-Chief have retracted this article because there appear to be image duplications in the Vimentin, CD44 and E-cadherin rows of Fig. 8F. The Editors-in-Chief therefore no longer have confidence in the integrity of the data in this article [1].

Xiaojun Fu does not agree to this retraction. Hongwang Song, Chenxing Wu and Shouwei Li have not responded to any correspondence from the publisher about this retraction.
